# Lessons learned by surveillance during the tail-end of the Ebola outbreak in Guinea, June-October 2015: a case series

**DOI:** 10.1186/s12879-017-2405-x

**Published:** 2017-04-24

**Authors:** Mory Keïta, Fatoumata Conté, Boubacar Diallo, Dieudonné Lufwa, Jacques Katomba, René Snacken, Raymond Pallawo, Aminata Tolno, Amadou Bailo Diallo, Mamadou Harouna Djingarey, Lorenzo Subissi

**Affiliations:** 1 World Health Organization (WHO), Conakry, Guinea; 2 Ministry of Health and Public Hygiene of the Republic of Guinea, Conakry, Guinea; 3 Ministry of Public Health of the Democratic Republic of Congo, Kinshasa, Democratic Republic of the Congo; 4 European Centre for Disease Prevention and Control (ECDC), Stockholm, Sweden; 5 National Agency for Health Security, Conakry, Guinea

**Keywords:** Ebola, Guinea, Surveillance, Contact tracing, Transmission, Outbreak, Case series, Epidemic

## Abstract

**Background:**

By the end of the 2013–2016 West African Ebola Virus Disease (EVD) outbreaks, a total of 3814 cases (probable and confirmed) and 2544 deaths were reported in Guinea. Clearly, surveillance activities aiming at stopping human-to-human transmission have been the breakthrough of EVD outbreak management, but their application has been at times easier said than done. This article presents five confirmed or probable EVD cases that arose in Conakry towards the end of the Guinea epidemic, which demonstrate flaws in surveillance and follow-up.

**Case presentation:**

For case 1, safe burial requirements were not followed. For cases 1 and 2, negative Polymerase Chain Reaction (PCR) results were interpreted as no infection. For the first case, the sample may have not been taken properly while for the second the disease was possibly at its early stage. Case 3 was stopped at a border health checkpoint and despite her high temperature she was allowed to continue the bus journey. For case 4, an oral swab sample was supposedly taken after death but could not be found for retrospective testing. Despite characteristic symptomatology, case 5 was not identified as a suspect case for as long as 3 weeks.

**Conclusion:**

In epidemic contexts, health systems must be able to track all samples of suspect cases and deaths, regardless of their laboratory results. Social mobilization in communities and training in health care facilities must be strengthened at the tail of an outbreak, to avoid the natural slackening of disease surveillance, in particular for long-lasting and deadly epidemics.

## Background

**Fig. 1:**
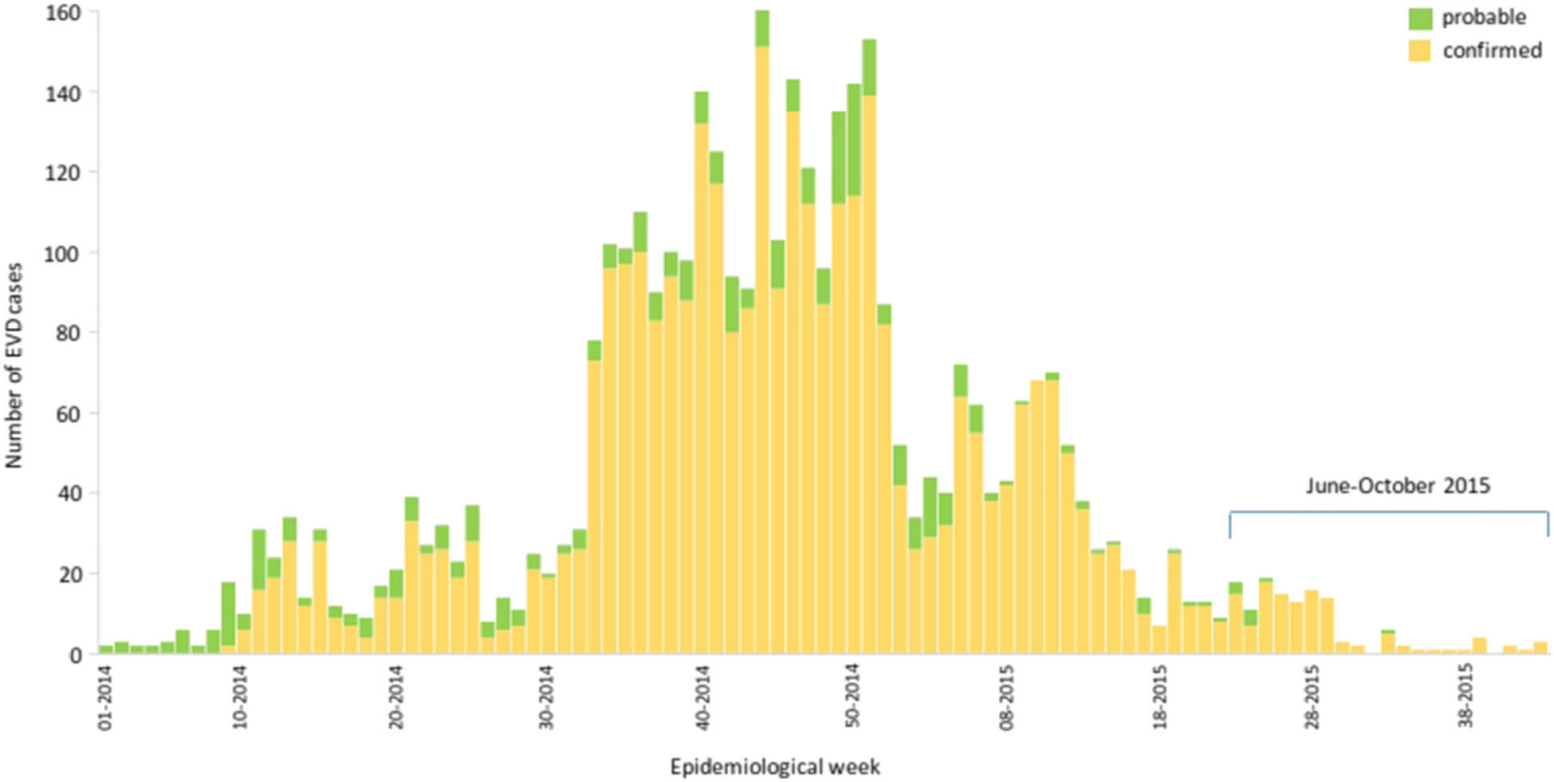
Epidemiological curve of Ebola virus disease in Guinea, January, 2014 to October, 2015

The 2013–2016 West African Ebola virus disease (EVD) epidemic has produced around 30,000 cases and 11,310 deaths. In Guinea, where the outbreak originated, there have been 3355 confirmed and 456 probable cases, including 2544 deaths [1]. The outbreak started in forested Guinea at the end of December, 2013 and quickly spread to urban settings including the capital, Conakry. By the end of October, 2015, the peak was reached (Fig. 1). From August, 2015, only sporadic cases were reported, mainly in Conakry and in the Forécariah prefecture. On December 29th, after two disease incubation periods (i.e. 42 days) from the hospital release of the last reported case, Guinea was declared Ebola-free.

This unprecedented outbreak created such a big global public health crisis that the WHO Director-General referred to it as “the greatest peacetime challenge that the United Nations and its agencies have ever faced” [2]. Monitoring and active surveillance during the outbreak used WHO case definitions [3]. An EVD confirmed case was an individual with laboratory confirmation of Ebola virus infection. The definition of ‘probable case’ was used to refer to a deceased person without any laboratory testing but with an epidemiological link to a confirmed case.

In August 2014, WHO drafted the Ebola Response Roadmap to set out the core strategy for stopping the outbreak and providing the basis for a significantly increased response [4]. This required a rapid scale-up of treatment facilities, burial capacity and behavioral changes, followed by the rapid scale-up of rigorous case finding, contact tracing and intense community engagement to interrupt residual EVD transmission chains.

Referring to this international guideline, a surveillance protocol was put in place in Guinea. It included: i) a mobile team of senior medical doctors assigned to evaluate on a daily basis possible cases reported by junior medical teams deployed in the field; ii) community- and health facility-based active surveillance aiming at early detection of EVD suspect cases; iii) rapid transfer of suspect cases to an Ebola treatment unit (ETU) for laboratory testing [5]; iv) identification and follow-up over 21 days (ie, the maximum EVD incubation period) of all contacts of those with a positive Ebola virus (EBOV) test; v) oral swabbing of all those individuals who died in the community for EBOV testing; vi) safe burial of all declared deaths, ensured by the Red Cross trained personnel; vii) temperature monitoring of travelers (using a non-contact thermometer) at check-points placed at the air, water and land borders of main cities, and viii) molecular surveillance by systematic genetic sequencing of EBOV positive samples. In addition, the use of rapid diagnostic tests [6], a ring approach to infection prevention and control [7] and ring vaccination [8] were implemented in the country during the later months, to accelerate progress towards the end of the outbreak.

Malfunctions and setbacks in the implementation of Ebola surveillance and public health protocols must be shared to guide and improve response and preparedness for future outbreaks, should they be due to EVD or another disease that needs similar surveillance. Here we report cases for which faults of the surveillance system may have slowed the process of bringing the EVD epidemic to a close.

## Methods

We chose all confirmed or probable EVD cases that arose in Guinea between June and October 2015 for which case management and surveillance have shown flaws. Reported information was taken from official WHO case investigation reports. Blood samples of living patients and oral swab samples of dead individuals were tested for EBOV by Polymerase Chain Reaction (PCR) in eight accredited facilities spread throughout the country.

## Case presentation

### Case 1

**Fig. 2:**
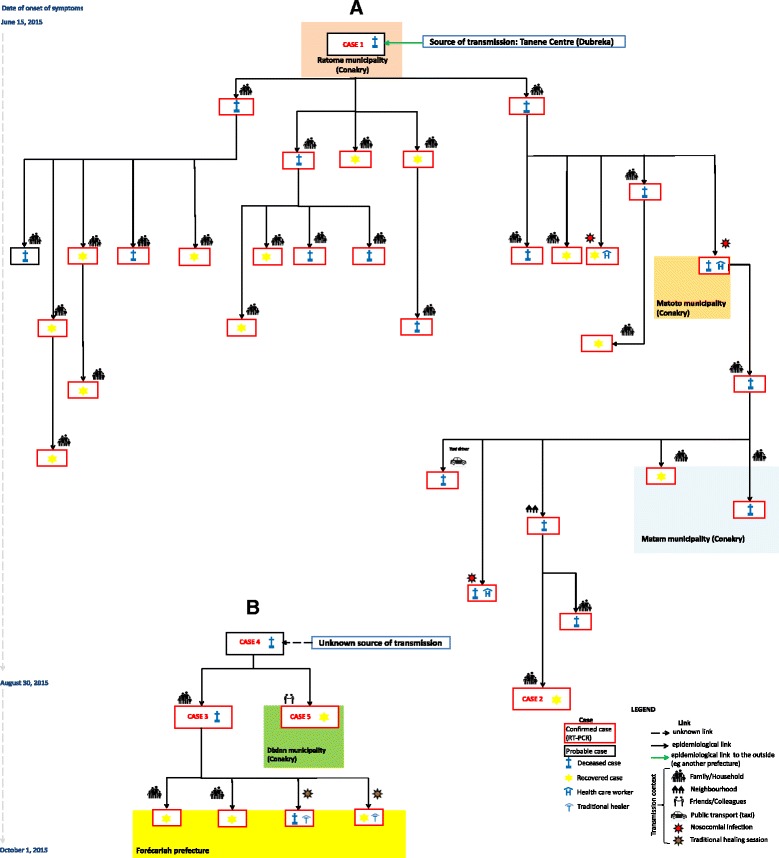
Chains of transmission of Ebola virus in Conakry, the capital of Guinea, and in a surrounding prefecture (Forécariah), from June to September 2015. **a** The chain of transmission initiated by Case 1 which lasted 3 months and resulted at least 30 confirmed and 2 probable cases, including 16 deaths. **b** A hidden chain discovered during the epidemiological investigation whose source remains unknown. This chain extended to the prefecture of Forécariah where it made 4 confirmed cases

A 49-year-old woman died in a national hospital in Conakry on June 19th, 2015 (Fig. 2a). In the previous 2 weeks, the woman had traveled to Tanene village, in the Dubreka prefecture, where EVD transmission was ongoing. As in all in-hospital deaths at that time, an oral swab sample was taken. It was negative for Ebola virus by PCR. The body was left to the family instead of being safely buried by the Red Cross, as required by the protocol in place. On July, 6th, two contacts of the woman - the sister and a close neighbor - died. The oral swab samples from both contacts tested positive for Ebola virus, and case 1 was therefore classified as a probable EVD case. The negative sample - which was still available – was retrospectively tested by another laboratory but gave invalid results because of lack of human DNA. This may indicate that the first test was invalid rather than negative.

### Case 2

**Fig. 3:**
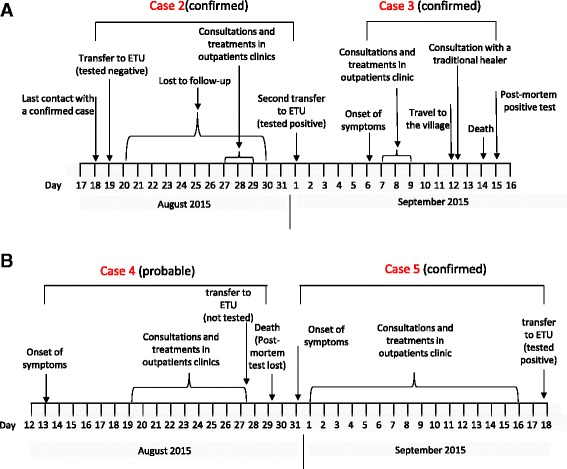
**a**) Timeline of events of cases 2 & 3 from 18th August to 15th September 2015. These two cases are confirmed. **b**) Timeline of events of cases 4 (probable) & 5(confirmed) from 13th August to 18th September 2015

A 13-year-old, who was a relative of one confirmed case and was therefore followed-up as a known contact, presented with fever, abdominal pain and asthenia to an outpatient clinic in Ratoma. She was sent to the ETU, where she initially tested negative for EBOV. Under the influence of some reluctant family members, she was lost to follow-up for 10 days. Her symptoms persisted and, with the help of other family members, she sought care. She was soon transferred to the ETU, where she tested positive for EBOV (Fig. 3a). Fortunately, she recovered few days later, and no additional cases are known to have been caused by this case.

### Case 3

A 12-year-old girl resident in Ratoma presented with diarrhea and vomiting to a large outpatient clinic, where she was treated for suspected malaria. She did not have an epidemiological link with any confirmed case. Despite her symptomatology, she was not considered an EVD suspect case. She travelled by bus to Forécariah a few days later. Few months earlier, to avoid reintroduction of EVD in the Forécariah prefecture, a health check-point at the prefecture border was set up. At the check-point, the girl’s temperature was repeatedly taken and found to be >38 °C. For unknown reasons, she was however allowed to continue her journey to her final destination. Once in Forécariah, she sought treatment from a traditional healer, who was infected when giving her care. She passed away 2 days later, and a post-mortem swab confirmed infection with EBOV (Fig. 3a). This case gave rise to 4 confirmed cases, including one death (Fig. 2b).

### Case 4

During the investigation of case 3, an earlier death in the family a 19-year-old woman was retrospectively considered as a probable case (Fig. 2b). The investigation revealed that she had presented with headache, asthenia, chills, abdominal pain and appetite loss and sought care in a main hospital in Conakry, from which she was referred to an ETU. Upon arrival, doctors did not deem her as an EVD suspect case and she was therefore not tested for EBOV. She was referred back to the hospital where she died 1 day later. Her corpse had routine oral swabbing for EBOV testing, but for unknown reasons those samples were neither tested nor registered and were missing when retrospectively sought (Fig. 3b). Her safe burial was ensured by the Red Cross, and no additional cases are known to have been caused by case 4.

### Case 5

A 23 year-old woman, who lived with cases 3 and 4 (Fig. 2b) presented asthenia, appetite loss, dysphagia and lower abdominal pain. She sought care in different secondary care facilities but was not evaluated for EBOV infection. Eventually, after having been symptomatic in the community for nearly 3 weeks, she was referred to the ETU where she tested positive for Ebola virus (Fig. 3b). A few days later she recovered and, to our knowledge, she did not infect other people.

## Discussion

As observed in previous Ebola outbreaks and in Liberia and Sierra Leone during the 2013–2016 outbreaks, strict control measures are effective for interrupting disease transmission [9, 10]. These measures focus on the early detection of cases and the comprehensive follow-up of contacts [11]. Efforts to find missing and unknown contacts are critical, but they were often hampered by a widespread distrust of health authorities in communities [12, 13]. Flaws were identified during the investigation of the last EVD cases detected in Conakry during the tail of the epidemic. Those flaws were mainly associated to lack of suspicion for EVD despite compatible clinical presentation. Other factors were insufficient effort to follow-up contacts and/or fight distrust of health authorities, failure to follow very demanding safe burial requirements and slackened controls at border health checkpoints. Misinterpretation of laboratory results may also have occurred. The reasons for the slackening of the surveillance system were not clearly identified; rumors that the outbreak was fading may have played a role similarly, after months of hard work, the fatigue of health care workers may have caused lapse of concentration.

Detailed information of all suspects’ cases that are tested for EBOV must always be stored in a database, to ensure quick traceability and avoid loss-to-follow-up of potential false negatives. Samples should be kept as long as possible, depending on the resources in place and the epidemiological situation in the country. Moreover, samples’ traceability should be guaranteed to allow retrospective testing. These databases may be anonymized to protect survivors’ identity and avoid stigma.

Once the peak of the epidemics is clearly over, supervision of control strategies in place must be enhanced. This is of great importance considering the risks of EVD flare-ups [13–17]. These strategies include health care-based and (whenever possible) community-based supervised surveillance, training of medical staff for detection of suspect cases and training for an appropriate and timely use of rapid diagnostic tests [18]. Because clinical evaluation may be misleading [19], definition of suspect case must have a high sensitivity, at some cost to specificity.

## Conclusions

To conclude, long-lasting and deadly epidemics such as the 2013–2016 West African outbreak are more exhausting and stressful to control and they may therefore be more susceptible to lapses in surveillance at their tail-end. Therefore, fine-tuning of the disease fighting machinery in place is critical when a country enters the last phase of an epidemic. In our opinion, this should include strengthening of social mobilization interventions in communities and continuous training of health care workers.

## Abbreviations

EBOV: Ebola virus
ETU: Ebola treatment unit
EVD: Ebola virus disease
PCR: Polymerase chain reaction
WHO: World Health Organization
